# Supplementary figures and images for: Identification and charactering of APX genes provide new insights in abiotic stresses response in Brassica napus
Source: PeerJ. 2022 Apr 5;10:e13166. doi: 10.7717/peerj.13166 (PMC8992642; doi:10.7717/peerj.13166)

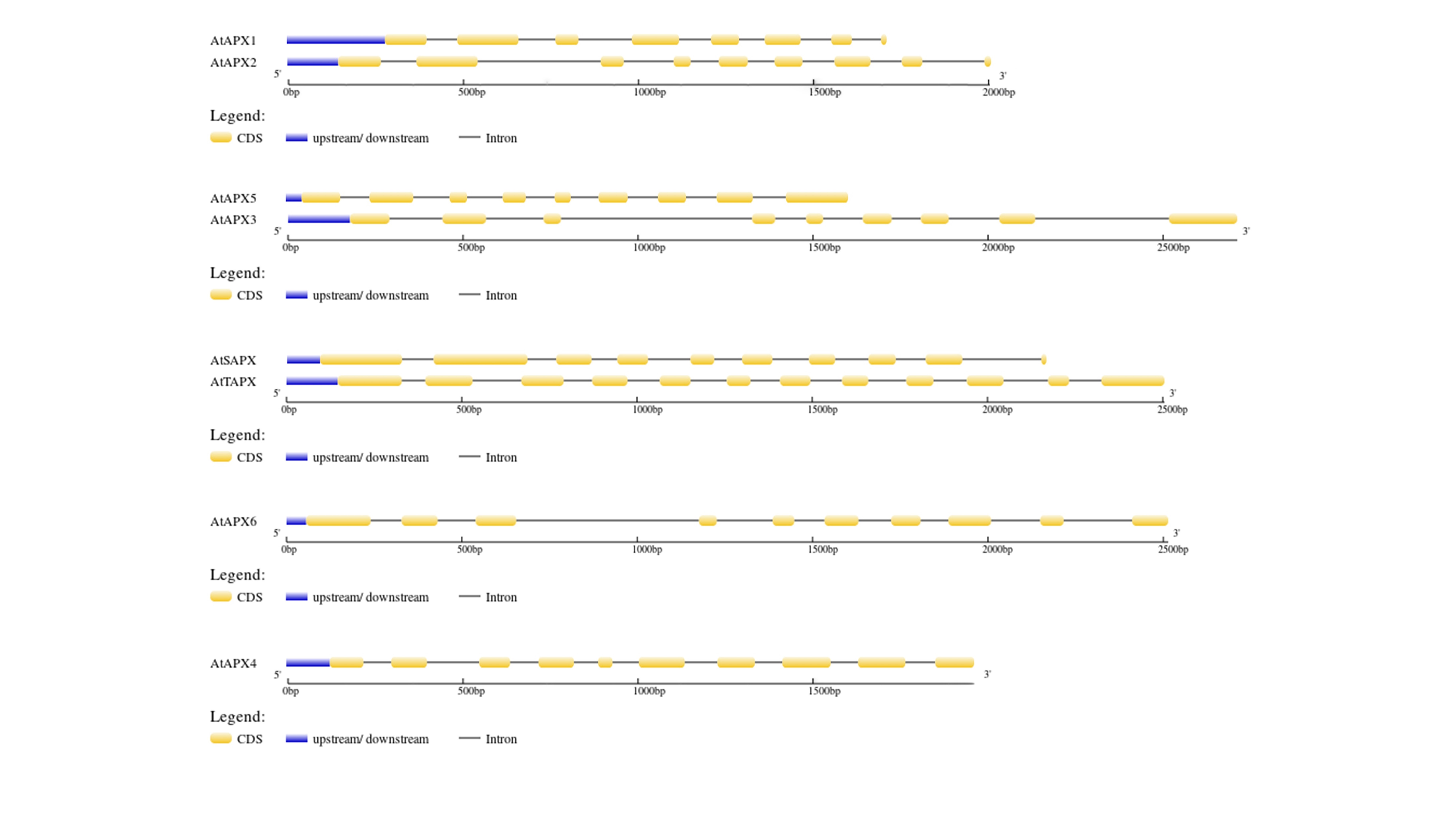

Supplement: Supplemental Information 3 — The gene structure of 26 APX genes was constructed by Gene Structure Display Server 2.0 ( http://gsds.cbi.pku.edu.cn/). Yellow boxes represented exons, black lines represented introns and blue boxes represented upstream or downstream. The sizes of exons can be estimated by the scale at bottom. [file peerj-10-13166-s003.jpg]

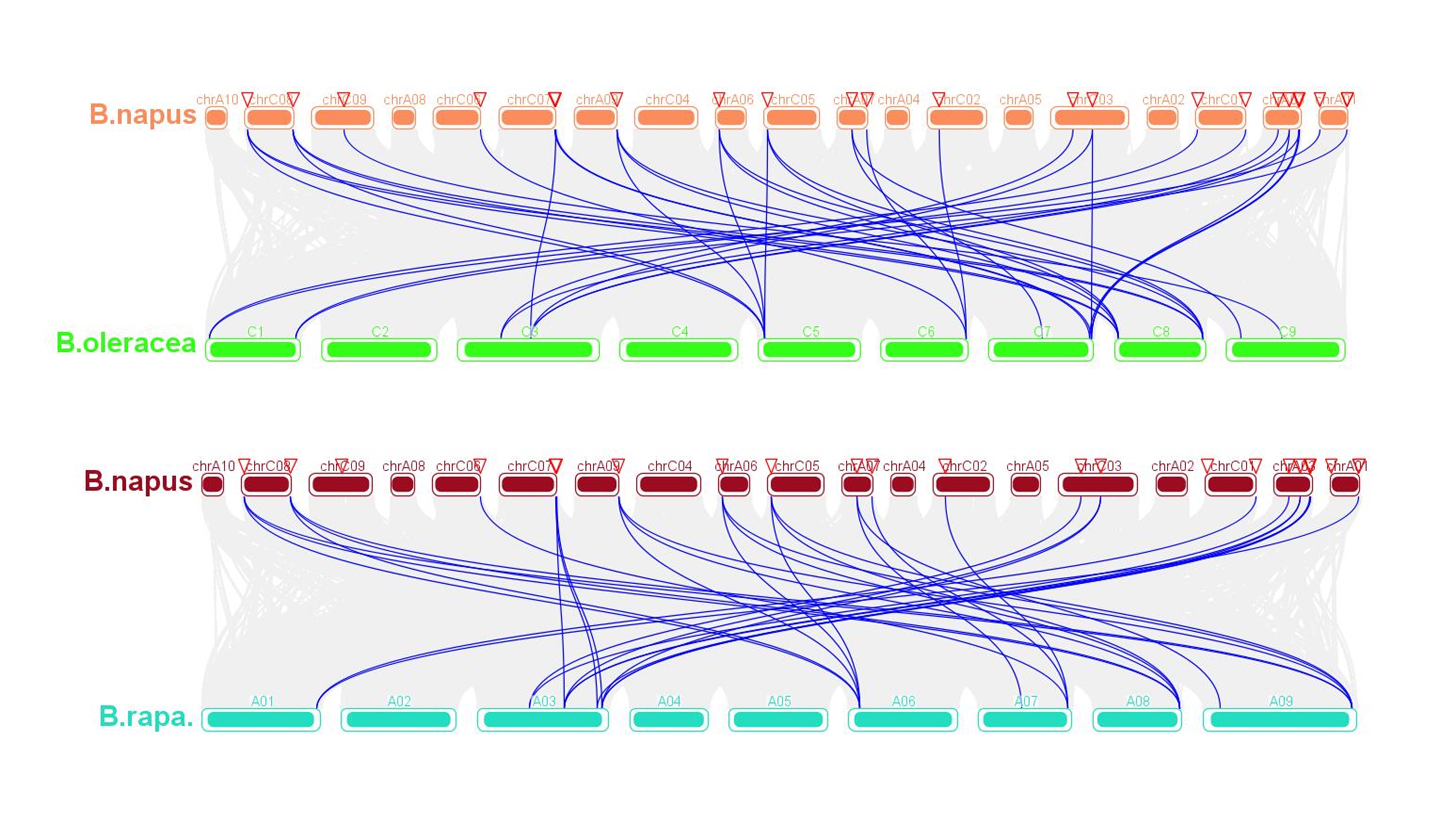

Supplement: Supplemental Information 4 — Described the synteny analysis of APX family between B. napus and its parents [file peerj-10-13166-s004.jpg]
